# Reproductive health of women with endometriosis: an improving educational intervention based on the planned behavior theory

**DOI:** 10.1186/s43043-023-00129-7

**Published:** 2023-02-23

**Authors:** Niusha Zandi, Zahra Behboodi Moghadam, Batool Hossein Rashidi, Masoumeh Namazi, Shima Haghani

**Affiliations:** 1 Department of Midwifery and Reproductive Health, School of Nursing and Midwifery, Tehran University of Medical Sciences, Tehran, Iran; 2 Valiasr Reproductive Health Research Center, Tehran University of Medical Sciences, Tehran, Iran; 3 Nursing Care Research Center, Iran University of Medical Sciences, Tehran, Iran

**Keywords:** Endometriosis, Education, Planned behavior theory, Reproductive health

## Abstract

**Background:**

Endometriosis is a chronic deliberating disease with devastating effects on reproductive health. The present study aimed to investigate the impact of education based on the theory of planned behavior (TPB) on the reproductive health of women with endometriosis. This research was a randomized controlled trial performed on 71 women with endometriosis (35 intervention and 36 control groups) referred to the infertility clinic of Imam Khomeini Hospital in Tehran, Iran. The educational intervention based on the structures of the TPB was performed in the intervention group in 4 sessions, weekly for 90–120 min. The demographic questionnaire, model constructs questionnaire, and endometriosis reproductive health questionnaire (ERHQ) in both groups were completed in 3 stages (before intervention, 4, and 8 weeks after the intervention). Data were analyzed using SPSS software version 24.

**Results:**

After the educational intervention, TPB values and overall reproductive health of women with endometriosis improved significantly in the intervention group (*p* < 0.05), while changes were not significant in the control group.

**Conclusion:**

The study results showed that education based on the TPB had positive effects on the reproductive health of patients.

**Trial registration:**

IRCT20120414009463N64. Registered 21 Jun 2021 - Retrospectively registered, http://www.irct.ir/trial/53341.

**Supplementary Information:**

The online version contains supplementary material available at 10.1186/s43043-023-00129-7.

## Introduction

Endometriosis is a chronic deliberating disease defined as the presence of endometrial glands and stroma outside the uterine cavity, which causes pelvic pain and infertility. It affects approximately 5-15% of women within their reproductive age. Endometriosis should be viewed as a public health problem with a major impact on the reproductive health of women as well as being a substantial economic burden [1–3]. Endometriosis has devastating effects on different aspects of reproductive health, including physical, psychosocial, social, and sexual health, and leads to extensive problems with infertility that harm everyday activities, marital relationships, and self-confidence and causes mental disorders [4].

Applying new strategies to improve behaviors and lifestyle modifications can be effective in maintaining and promoting the reproductive health of women with endometriosis [5, 6]. Establishing a suitable model for behavior change is a necessity for doing an effective intervention [6]. In this study, we chose the theory of planned behavior to improve the reproductive health of women with endometriosis, as it has been used in many studies [7–10]. The theory of planned behavior (TPB) provides a systematic framework for health education. This theory was first developed by Icek Azjen et al., and it is categorized by the following: (1) attitude: an individual's positive or negative evaluation of self-performance of the particular behavior; (2) subjective norms: an individual's perception about the particular behavior, which is influenced by others, such as parents, spouse, friends and, teachers; (3) behavioral intention: the decision and willing of the individual to behave in a particular manner; and (4) behavior: an individual’s response in a specific situation to a given target [7].

The purpose of this study was to investigate the effect of education based on the theory of planned behavior in order to improve the reproductive health of women with endometriosis.

## Methods

This randomized controlled trial was carried out among 71 women with endometriosis (35 intervention and 36 control groups) referred to the infertility clinic in Imam Khomeini Hospital in Tehran from December 2020 to August 2021. All eligible women were included in the study, and participants were selected by simple random sampling method.

The inclusion criteria were being 15–45 years old, married and living with husband, confirmed diagnosis of endometriosis through laparoscopy, volunteering to participate in the study, not having a history of psychological problems or chronic diseases, and ability to use the internet. The exclusion criteria were being absent in two or more sessions. The permuted block randomization was conducted with a computer-generated list. The type of treatment was placed in sealed envelopes and the assignment to intervention and control groups was performed by the out-of-study midwife.

Informed consent was obtained from each participant. The study was approved by the Ethics Committee of Tehran University of Medical Sciences (IR.TUMS.FNM.REC.1399.148) on 28/12/2020. It was registered in the Iranian Registry of Clinical Trial with the number IRCT20120414009463N64.

### Sample size

The sample size was calculated using the following formula based on the effect size and Cohen Statistical Power Analysis [11]:

$$n=\frac{2\times {\left({z}_{1-{}^{\alpha }\!\left/ \!{}_{2}\right.}+{z}_{1-\beta}\right)}^2}{E.{S}^2}=\frac{2\times {\left(1.96+0.84\right)}^2}{(0.7)^2}=34$$

$${z}_{0.975}=1.96$$

$${z}_{0.8}=0.84$$

**Fig. 1:**
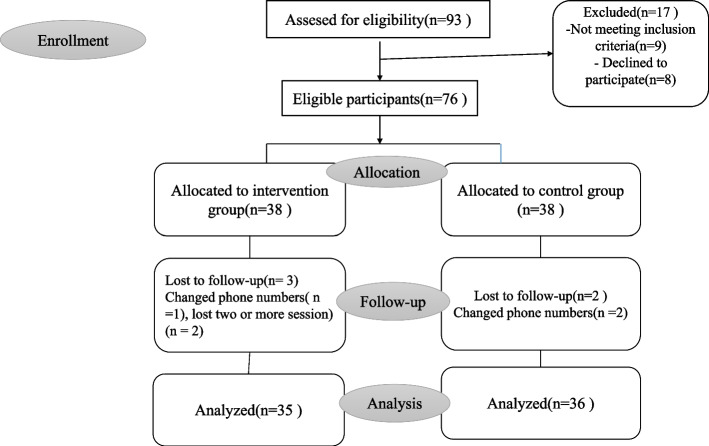
Study flowchart

With a confidence interval of 95% and power of 80%, the number of samples was calculated to be 34 for each group. Anticipating some dropout cases, the sample size was calculated to be 38 in each group. After follow-up, 35 patients in the intervention group and 36 patients in the control group were compared. A study flowchart is presented in Fig. 1.

### Data collection

The data were gathered in 3 stages (before the intervention, 4, and 8 weeks after the intervention) through the following questionnaire:

- Demographic questionnaire includes age, husband’s age, education, job, economic status, smoking, history of medicine use, number of children, type of childbirth, disease duration.
- Model constructs questionnaire: the questionnaire was developed based on the TPB. We designed a questionnaire according to scientific resources and expert comments, including the professors of midwifery, reproductive health, and gynecology. The questionnaire consisted of four subscales: questions for knowledge measurement (11 items), items on attitude measurement (6 items), subjective norms (8 items), and behavioral intention (1item).

The knowledge subscale assessed the participants’ knowledge about impact of endometriosis on sexual health, reproductive health, sexual function, desire to have children, fertility and infertility, shame and guilt feeling, impact of drug treatment on sexual function, need for sexual counseling and valid information. The knowledge subscale was assessed using a 5-point Likert scale: (5) extremely agree, (4) slightly agree, (3) neutral, (2) not agree, (1) extremely disagree. The total subscale score ranged from 11 to 55, where a higher score pointed out a higher level of knowledge. The attitude subscale assessed the participants’ attitude about sexual relationship, sexual desire, fertility problems, sexual satisfaction, having enough information about sexual health and sexual intimacy. The attitude subscale was designed based on a 5-point Likert scale on an agreement scale ranging from (1) to (5). The total subscale score ranged from 6 to 30 scores. The higher the score, the more positive the attitude toward the reproductive health. The subjective norms subscale assessed the social relations of women with their spouse and family members, awareness of family members from disease, spouse’s behavior regarding disease, impact of friends and family members on sexual relationship. This subscale was assessed using a 2-point scale: (1) no and (2) yes. The total subscale score ranged from 8 to 16 scores. The greater the score, the more effective the subjective norms were on women’s adherence to reproductive health. The behavioral intention subscale assessed the tendency of women to go to sexual and fertility counseling centers in the next 6 months. It was assessed using a 2-point scale: (1) no and (2) yes. The total subscale score ranged from 1 to 2. The higher the score, the stronger the women’s intention to adhere to reproductive health.

The validity of the questionnaire was determined by two methods of face validity and content validity. For determining face validity, the scale was distributed among 10 women with endometriosis and asked to identify any difficulties with interpretations of the sentences (understanding phrases, expressions, and words). Then the item impact method was used to determine the importance of each phrase. Finally, the respondents scored the items using a Likert 5-point scale, from “not at all important (score1)” to “extremely important (score5)”. The impact sores of the items were calculated, and the items with an impact score of ≥ 1.5 were included [12].

For assessing content validity, we asked ten experts of midwifery, reproductive health, and gynecology to review the items of the questionnaire. The content validity ratio (CVR) and content validity index (CVI) were calculated for each item. The content validity of items was calculated; in the Lawshe table, an acceptable content validity ratio (CVR) value for ten experts is 0.62 [13]. Then, the content validity index (CVI) was used to ensure that the items were properly designed. The method of Waltz and Bausell was used to assess the CVI, where an item with a CVI of > 0.79 is acceptable [14]. The total content validity index (CVI) in the “relevancy”, “simplicity”, and “clarity” respectively were 83.4, 91.74, and 90.52.

The reliability of the questionnaire was evaluated through internal consistency (α = 0.91) and test-retest (*r* = 0.82). Cronbach’s alpha was used for assessment of the reliability. A Cronbach’s alpha value between 0.8−0.9 shows high internal consistency, and an alpha value greater than 0.70 indicates desired internal consistency [15].

- Endometriosis reproductive health questionnaire (ERHQ): It is a valid and reliable questionnaire developed by Namazi et al. (2021). The questionnaire contains 35 items on five factors. The first factor, “physical problems,” including nine items, and the second factor, “psychological problems,” including 12 items. The third and fourth factors (counteracting strategies and the instability of marital life, respectively) consisted of 6 and 8 items. Each question includes a 5-point Likert scale: (5) extremely agree, (4) slightly agree, (3) neutral, (2) not agree, (1) extremely disagree, so the minimum total number of the questionnaire is 35, and the maximum is 175. The reliability of the questionnaire, according to Cronbach’s alpha, is 0.809, and the external reliability, as evaluated by the test-retest method and the intraclass correlation, is 0.825 [16].

### Intervention

The content of the intervention was prepared based on using validated scientific resources. Due to the COVID-19 pandemic, the educational content was provided online, and the questionnaires were completed online. The intervention group was divided into groups of four to eight. Four sessions of 90–120 min were held once a week. The first session was designed to increase the participants ‘knowledge about endometriosis definition, epidemiology, risk factors, and prevention. The second session targeted the explanation of sign and symptoms, diagnostic methods, management strategies, and lifestyle modifications. The third session concerned with correcting any misinformation about endometriosis, infertility, reproductive health, and sexual function to foster positive attitudes and beliefs among women about endometriosis. To change attitudes, measures such as discussion in small groups and sharing experiences with others, and learning from each other were done. The researcher had the role of correcting misconceptions. The fourth session aimed to address the subjective norms that affect the women’s intention for behavior. The key person was identified for each participant and added to the Skype environment to attend the meetings. Facilitating and inhibiting factors were discussed in these sessions. For example, the costs of treatment and travel to the centers providing these services and possible obstacles and their appropriate solutions were discussed. The teaching strategies were included lectures, group discussion, and brainstorming. At the end of each session, a summary of the content and feedback was provided.

For the control group, relevant questionnaires were completed, then they left for the routine hospital care, and revaluation was done after 4 and 8 weeks. The educational content was provided after the end of the 8 weeks of enrolment in the study.

### Statistical analysis

Data were analyzed using SPSS software version 24 and statistical exams, including the repeated-measure analysis, *t* tests, and chi-square tests (significance level *P* < 0.05).

### Ethics

The ethics committee of Tehran University of Medical Sciences approved the study (IR.TUMS.FNM.REC.1399.148) on 28/12/2020. All participants signed informed consent. All methods were performed in accordance with the relevant guidelines and regulations.

## Results

The results showed no significant differences between the control and intervention groups in terms of demographic variables. Independent-sample *t* test results showed that there were no significant differences between the intervention and control groups regarding the mean age, husband’s age, and disease duration. Chi-square tests also showed no significant difference between education, job, economic status, smoking, history of medicine use, number of children, and type of childbirth in both the intervention and control groups (*P* > 0.05) (Table 1).

**Table 1:** Socio-demographic characteristics of the participants

	Groups		***P***
Variable	Intervention	Control	
Age (Mean ± S.D.)	35.50±3.44	33.70±2.24	0.730
Husband’s age (Mean ± S.D.)	37.22±3.71	37.25±3.64	0.960
Disease duration (Mean ± S.D.)	8.1±0.13	7.89±0.24	0.420
Education			
Elementary	22.50%	34.20%	0.690
Diploma	58.50%	51.50%	
Bachelor or more	19%	14.3%	
Job			
Housewife	61.10%	65.70%	0.680
Employed	38.90%	34.30%	
Economic status			
Poor	11.50%	8.50%	0.930
Medium	75%	77%	
Good	13.50%	14.50%	
Smoking			
Yes	14%	14.50%	0.960
No	86%	85.50%	
History of medicine use			
Yes	86.50%	83%	0.700
No	13.50%	17%	
Number of children			
0	66%	62%	0.760
1	25%	29.50%	
Two or more	9%	8.50%	
Type of childbirth			
NVD	48.30%	49.53%	0.941
C.S.	51.70%	50.47%	

The results showed no significant differences between the control and intervention groups in terms of knowledge, attitude, subjective norms, and behavioral intention before the intervention (*P* > 0.05), but 4 and 8 weeks after the intervention, the mean of these variables in the intervention group were greater than the ones in the control group (*P* < 0.05) (Table 2).

**Table 2:** Comparison of the planned behavior theory components between intervention and control groups

**Knowledge**	**Before Intervention** **Mean ± S.D.**	**Four weeks after the Intervention** **Mean ± S.D.**	**Eight weeks after the Intervention** **Mean ± S.D.**	P*
Intervention group	27.55±1.6	37.67±2.28	42.13±2.28	<0.001
Control group	26.62±2.18	27.48±2.69	28.62±3.06	0.48
P**	0.46	<0.001	<0.001	
**Attitude**	**Before Intervention** **Mean ± S.D.**	**Four weeks after the Intervention** **Mean ± S.D.**	**Eight weeks after the Intervention** **Mean ± S.D.**	P*
Intervention group	18.25±1.90	20.44±2.23	22.02±1.70	<0.01
Control group	18.31±1.81	18.32±1.90	18.32±2.22	0.49
P**	0.885	<0. 01	<0.001	
**Subjective norms**	**Before Intervention** **Mean ± S.D.**	**Four weeks after the Intervention** **Mean ± S.D.**	**Eight weeks after the Intervention** **Mean ± S.D.**	P*
Intervention group	12.02±3.80	15.25±1.31	15.71±3.66	<0.01
Control group	12.57±4.01	12.71±1.29	12.88±2.54	0.29
P**	0.564	<0. 01	<0.001	
**Behavioral intention**	**Before Intervention** **(yes percent)**	**Four weeks after the Intervention** **(yes percent)**	**Eight weeks after the Intervention** **(yes percent)**	P*
Intervention group	33.3%	34.1%	35.2%	<0.01
Control group	34.3%	29.5%	26%	0.39
P***	0.932	<0. 001	<0.001	

*Notes*: *Derived from repeated-measure analysis; **derived from *t*-tests, ***derived from χ2

The results showed no significant differences between the control and intervention groups in terms of reproductive health before the intervention (*P* > 0.05). But 4 and 8 weeks after the intervention, the reproductive health of women with endometriosis in the intervention group improved significantly (*P* < 0.05) (Table 3).

**Table 3:** Comparison of women’s reproductive health with endometriosis between intervention and control groups

	Before Intervention Mean ± S.D.	Four weeks after the Intervention Mean ± S.D.	Eight weeks after the Intervention Mean ± S.D.	P*
**Intervention group**	85.41±6.2	124.91±8.9	125.22±9.69	<0.001
**Control group**	87.51±12.96	84.65±11.14	82.62±18.55	0.342
P**	0.385	<0.001	<0.001	

*Notes*: *Derived from repeated-measure analysis; **derived from *t*-tests

## Discussion

The results of this study showed that education based on the theory of planned behavior improves the reproductive health of women with endometriosis. Accordingly, when women with endometriosis get adequate knowledge about their disease and are presented with a positive attitude toward it, they will be determined to change their behaviors.

Endometriosis can adversely affect the reproductive health of patients, including physical suffering, instability of marital life, psychological disorder, and disruption in social life. Physically, endometriosis causes menstrual disorder, including severe bleeding, spotting, and irregular menstruation. Disabling pelvic pain is another physical problem that most of patients suffer from it—psychological problems such as frustration, a feeling of repeated failure, and isolation. Instability of marital life is one of the most important problems of women with endometriosis that can occur in the forms of emotional tension with the spouse and sexual dissatisfaction. Endometriosis complications such as infertility, dyspareunia, depression, and anxiety can be a threatening factor in the emotional intimacy between couples. Sexual problems such as dyspareunia, loss of libido, inability to achieve orgasm and reduction in the number of intercourses due to spotting and dyspareunia are common among women with endometriosis [2, 4, 16, 17]. So, the need for effective intervention in order to improve their reproductive health is felt. The results of the present study show that a health-education program based on the planned behavior theory improves the reproductive health of these patients.

The pre-intervention baseline survey showed that women’s’ knowledge, attitude, subjective norms, and behavioral intention about reproductive health were inadequate. Significant changes in total variables conclude that education is useful and practical in changing reproductive health behaviors in women with endometriosis after follow-up. The TPB-based educational intervention improved all dimensions of reproductive health of women with endometriosis.

Evaluation of the impact of the education intervention on participants’ knowledge indicated an overall improvement in scores. In a study by Jalambadani et al. (2018), training has been effective in encouraging iron supplementation in pregnant women [7]. The results of our study indicated a statistically significant difference in the scores of attitude in the experimental group before and after the educational intervention, consistent with another studies on reducing the high-risk behaviors related to sexual and reproductive health in adolescent girls [18], predicting adolescent condom use [19], and emergency contraception use among teenagers [20]. This attitude is a critical factor that encourages and motivates people to adopt behaviors. Therefore, it is necessary for intervention planners and policymakers to rely on the fundamental change in people’s attitudes by effective methods that motivate healthy behaviors [14]. In line with some studies [18, 21–23], subjective norms significantly increased in our study. However, another study did not confirm a significant increase in subjective norms due to the intervention [24, 25]. In the study by Jemmott III et al. (2007), subjective norm was not predictor of intention to use condoms among Xhosa adolescents in South Africa. The authors believe that subjective norms have somewhat weaker relations to intention than do attitudes and perceived behavioral control [24]. In another study by Gredig et al. (2006), subjective norm was not predictor of intention to use condoms in sexual encounters with new partners. A possible reason could be that subjective norm in men is lower than subjective norm in women [25].

In addition, a significant increase in the mean score of behavioral intention was observed in the experimental group, emphasizing the effectiveness of the intervention implementation, in line with other studies [26, 27]. In TPB, behavioral intention is regarded as the main behavior predictor. Intentions are as motivational factors affecting behavior and indicating the severity of individuals' motivation and efforts to perform a task. The more strong the behavioral intention, the more probable it is to do or change behavior [18]. Educational intervention could positively affect the women's behavioral intention for reproductive health promotion.

This study had some limitations. One was the short follow-up. Future studies need to follow women for longer. Also, because of the COVID-19 pandemic, the educational content was provided online, and face-to-face education was impossible. Finally, it seems that a bigger sample is needed to reach firm conclusions.

## Conclusion

The results of the study showed that education based on the theory of planned behavior had positive effects on the reproductive health of women with endometriosis.

## Supplementary Information

**Additional file 1.** Endometriosis reproductive health questionnaire (ERHQ).

**Additional file 2.** Model constructs questionnaire.

## Data Availability

The datasets used and/or analyzed during the current study are available from the corresponding author on reasonable request.

## Abbreviations

TPB: Theory of planned behavior
ERHQ: Endometriosis reproductive health questionnaire
CVI: Content validity index
CVR: Content validity ratio
