# Supplementary material for: Reproductive health of women with endometriosis: an improving educational intervention based on the planned behavior theory
Source: Middle East Fertil Soc J. 2023 Feb 23;28(1):4. doi: 10.1186/s43043-023-00129-7 (PMC9947440; doi:10.1186/s43043-023-00129-7)
Supplement: Supplementary file 1 — Additional file 1. Endometriosis reproductive health questionnaire (ERHQ). [file 43043_2023_129_MOESM1_ESM.docx]

| Number | Questions | Extremely agree | Slightly agree | Neutral | Not agree | Extremely disagree |
| --- | --- | --- | --- | --- | --- | --- |
| 1 | I have pelvic and abdominal pain. |  |  |  |  |  |
| 2 | Infertility caused by disease annoys me. |  |  |  |  |  |
| 3 | I have heavy bleeding with periods. |  |  |  |  |  |
| 4 | Pain with periods is intolerable for me. |  |  |  |  |  |
| 5 | I have to overuse painkillers with  periods to relief pain. |  |  |  |  |  |
| 6 | I am unable to work with periods. |  |  |  |  |  |
| 7 | I have to go to bed/lie down because  of the pain. |  |  |  |  |  |
| 8 | I have trouble sleeping because of the pain. |  |  |  |  |  |
| 9 | I have referred many doctors to cure disease. |  |  |  |  |  |
| 10 | I feel frustrated that the doctor(s) cure my disease. |  |  |  |  |  |
| 11 | I am worried about infertility caused  by endometriosis. |  |  |  |  |  |
| 12 | I feel I am different from others. |  |  |  |  |  |
| 13 | I compare myself with others because of disease. |  |  |  |  |  |
| 14 | I have lost less self-confidence. |  |  |  |  |  |
| 15 | I feel bad or short-tempered because of disease. |  |  |  |  |  |
| 16 | I feel sick. |  |  |  |  |  |
| 17 | I am worried about recrudesce of disease. |  |  |  |  |  |
| 18 | I am afraid my disease become  malignant. |  |  |  |  |  |
| 19 | I am worried my children are affected  by the disease in the future. |  |  |  |  |  |
| 20 | I feel deficient because of infertility. |  |  |  |  |  |
| 21 | I am worried about the impact of  endometriosis on the intestine. |  |  |  |  |  |
| 22 | Heat soothes my pain with periods. |  |  |  |  |  |
| 23 | I use complementary medicine to relieve pain. |  |  |  |  |  |
| 24 | I feel better when I go with friends. |  |  |  |  |  |
| 25 | I feel better when I go with friends. |  |  |  |  |  |
| 26 | I am looking to achieve more information about endometriosis. |  |  |  |  |  |
| 27 | Working helps me to forget my disease. |  |  |  |  |  |
| 28 | I feel regret about marriage because  of endometriosis. |  |  |  |  |  |
| 29 | I dispute with my partner because of  endometriosis. |  |  |  |  |  |
| 30 | I have pain during or after sexual activity. |  |  |  |  |  |
| 31 | I avoid sexual activity because of pain. |  |  |  |  |  |
| 32 | I feel hopeless because of the lack of sexual orgasm. |  |  |  |  |  |
| 33 | I do sex just because of my partner. |  |  |  |  |  |
| 34 | I am not willing to have sex with my partner. |  |  |  |  |  |
| 35 | I have fewer sexual activity because of disease |  |  |  |  |  |
