# Supplementary material for: Reproductive health of women with endometriosis: an improving educational intervention based on the planned behavior theory
Source: Middle East Fertil Soc J. 2023 Feb 23;28(1):4. doi: 10.1186/s43043-023-00129-7 (PMC9947440; doi:10.1186/s43043-023-00129-7)
Supplement: Supplementary file 2 — Additional file 2. Model constructs questionnaire. [file 43043_2023_129_MOESM2_ESM.docx]

**Model constructs questionnaire**

1) The knowledge subscale

| Number of questions | question | extremely agree | slightly agree | neutral | not agree | extremely disagree |
| --- | --- | --- | --- | --- | --- | --- |
| 1 | The sexual health of partners changes because of endometriosis. |  |  |  |  |  |
| 2 | The reproductive health of partners changes because of endometriosis. |  |  |  |  |  |
| 3 | The desire to have children changes because of endometriosis. |  |  |  |  |  |
| 4 | The sexual function of partners changes because of endometriosis. |  |  |  |  |  |
| 5 | Endometriosis impacts on couples' fertility. |  |  |  |  |  |
| 6 | Drug treatments have destructive effects on sexual function and sexual health. |  |  |  |  |  |
| 7 | Endometriosis causes women to feel shame and guilty during sex. |  |  |  |  |  |
| 8 | There are enough and valid information about the sexual health and fertility of women with endometriosis. |  |  |  |  |  |
| 9 | In addition to medicinal and palliative treatments, there should be enough attention to sexual health of couples. |  |  |  |  |  |
| 10 | Sexual problems of endometriosis can lead to infertility. |  |  |  |  |  |
| 11 | During the sexual and fertility consultations, the presence of the partner is needed. |  |  |  |  |  |

2) The attitude subscale

| Number of questions | question | extremely agree | slightly agree | neutral | not agree | extremely disagree |
| --- | --- | --- | --- | --- | --- | --- |
| 1 | Endometriosis can impact on sexual relationship. |  |  |  |  |  |
| 2 | Even with endometriosis, I can have sexual desire. |  |  |  |  |  |
| 3 | Endometriosis causes fertility problems. |  |  |  |  |  |
| 4 | Endometriosis reduces sexual satisfaction. |  |  |  |  |  |
| 5 | Having enough information improves sexual health. |  |  |  |  |  |
| 6 | Having a satisfying sexual relation causes more intimacy between partners. |  |  |  |  |  |

3) The subjective norms subscale

| Number of questions | question | Yes | No |
| --- | --- | --- | --- |
| 1 | Is your social relation with your family members good? |  |  |
| 2 | Is your social relation with your spouse's family members good? |  |  |
| 3 | Does your spouse understand your situation and have a good relationship with you? |  |  |
| 4 | Has your spouse changed her behavior towards you after being aware of your disease? |  |  |
| 5 | Do your family members aware of your disease? |  |  |
| 6 | Among the people who have the most influence on you, does communication with them affect your sexual relationship with your spouse? |  |  |
| 7 | Do your families affect your sexual relationship with your spouse? |  |  |
| 8 | Do your friends affect your sexual relationship with your spouse? |  |  |

4) The behavioral intention subscale

| Number of questions | question | Yes | No |
| --- | --- | --- | --- |
| 1 | Are you planning to go to sexual and fertility counseling centers in the next 6 months? |  |  |
